# Transcranial static magnetic stimulation modulates sensory interhemispheric inhibition as revealed by somatosensory evoked potentials and high-frequency oscillations

**DOI:** 10.1038/s41598-026-52100-x

**Published:** 2026-05-11

**Authors:** Yuki Tanaka, Aoki Takahashi, Kodai Minami, Kenta Oguma, Nodoka Shimizume, Yusuke Shinozaki, Isamu Ozaki, Tatsunori Watanabe

**Affiliations:** 1 Graduate School of Health Sciences, Aomori University of Health and Welfare, 58-1 Mase, Hamadate, Aomori 030-8505 Japan; 2 Department of Rehabilitation Sciences, Hirosaki University of Health and Welfare, Hirosaki, Aomori Japan; 3 Department of Advanced Technology in Medicine, Institute of Biomedical Engineering, Institute of Science Tokyo, Tokyo, Japan; 4 Waseda Institute for Sport Sciences, Waseda University, Tokorozawa, Saitama Japan

**Keywords:** Transcranial static magnetic stimulation, Plasticity, Event-related potentials, Non-invasive brain stimulation, Interhemispheric interaction, Somatosensory cortex, Neuroscience, Physiology

## Abstract

Transcranial static magnetic stimulation (tSMS) is a non-invasive neuromodulation technique that reduces cortical excitability. Although tSMS has been reported to attenuate interhemispheric inhibition (IHI) within the motor cortex, its effects on IHI within the primary somatosensory cortex (S1) remain unclear. The purpose of this study was to determine whether tSMS over the S1 modulates somatosensory IHI using a paired somatosensory evoked potentials (pSEPs) paradigm. In a randomized crossover design, twenty-five healthy young adults received either tSMS or sham stimulation over the right S1 (C4 in the international 10–20 system) for 20 min. SEPs were elicited by a test stimulus delivered to the right median nerve, preceded by a conditioning stimulus (CS) delivered to the left median nerve at an interstimulus interval of 10 ms. Recordings were obtained before, immediately after, and 20 min after stimulation. In addition to the N20 and P27 SEP components, high-frequency oscillations (HFOs) were extracted and classified into early (eHFOs) and late (lHFOs) components relative to the N20 peak latency. TSMS over the right S1 significantly reduced CS-induced suppression of lHFOs in the left S1 immediately after stimulation, whereas eHFOs and N20/P27 components remained unchanged. Given that N20/P27 components, eHFOs, and lHFOs are thought to reflect activity in Brodmann area 3b of the S1, thalamocortical input, and GABAergic interneuron activity, respectively, these findings are consistent with the possibility that tSMS modulates transcallosal interactions involving intracortical inhibitory processing within the somatosensory system.

## Introduction

The bilateral hemispheres exert mutual inhibition via transcallosal fiber, a phenomenon known as interhemispheric inhibition (IHI)^1,2^. From a clinical perspective, IHI between the bilateral primary motor cortices (M1)^3^ has been reported to contribute to motor recovery in patients with stroke. Specifically, excessive inhibition from the non-lesioned hemisphere to the lesioned hemisphere has been associated with impaired performance of the paretic hand^4,5^. Similarly, IHI between the bilateral primary somatosensory cortices (S1) can also be disrupted after stroke, resulting in an asymmetric interhemispheric interaction^6,7^. Importantly, greater interhemispheric asymmetry in the S1 has been linked to poorer neurological status and more severe motor impairment^8^. Together, these findings suggest that imbalanced interhemispheric interactions within motor and somatosensory systems may represent important therapeutic targets for stroke rehabilitation^8,9^.

Non-invasive brain stimulation (NIBS) techniques, including repetitive transcranial magnetic stimulation (rTMS)^10,11^ and transcranial direct current stimulation (tDCS)^12^, have been shown to modulate IHI between the bilateral M1. Clinically, low-frequency rTMS^13–16^ and cathodal tDCS^17^, both of which reduce cortical excitability, have been reported to facilitate functional recovery in patients with stroke when applied over the non-lesioned M1. In addition to rTMS and tDCS, transcranial static magnetic stimulation (tSMS), in which a small but strong neodymium magnet is placed on the scalp, has been shown to decrease IHI from the stimulated M1 to the contralateral, non-stimulated M1^18^. Furthermore, tSMS applied over the non-lesioned M1, in combination with rehabilitation, has been reported to significantly improve manual dexterity in the paretic hand in patients with stroke^19^. Beyond the motor system, several studies have demonstrated that NIBS can also modulate IHI between the bilateral S1. For example, 1-Hz (low-frequency) rTMS over the left S1 increased the N20–P27 amplitude of somatosensory evoked potentials (SEPs) recorded from the contralateral (right) S1, reflecting activity in Brodmann area 3b of the S1, in healthy adults^20^. Similarly, 1-Hz rTMS applied over the non-lesioned S1 improved somatosensory function in patients with stroke^21^. However, it remains unclear whether tSMS applied over the S1 can modulate somatosensory IHI.

IHI between the S1 has been examined using paired somatosensory evoked potentials (pSEPs) elicited by bilateral median nerve stimulation at predefined interstimulus intervals (ISIs)^22–24^. In this paradigm, a conditioning stimulus (CS) delivered to the median nerve on one side precedes a test stimulus (TS) delivered to the contralateral median nerve, and the SEPs evoked by the TS are typically suppressed, reflecting interhemispheric interactions between bilateral S1. Using this approach, Ragert et al.^22^ and Brodie et al.^23^ reported significant suppression of the TS-evoked N20 amplitude at specific ISIs. Similarly, Norata et al.^24^ demonstrated significant reductions in the TS-evoked N20 and P25 components of SEPs, as well as high frequency oscillations (HFOs) superimposed on the N20 component. Notably, suppression was evident for late HFOs (lHFOs), which occur after the N20 peak and are thought to reflect activity of GABAergic interneurons in area 3b, whereas early HFOs (eHFOs), which occur before the N20 peak and are associated with action potentials of thalamocortical fibers, were relatively unaffected. These findings indicate that the pSEPs paradigm provides a useful framework for assessing inhibitory interhemispheric interactions within the S1. However, it should be noted that this paradigm does not provide a direct measure of interhemispheric inhibition, but rather reflects the processing of the test response under the influence of a preceding CS, likely mediated by transcallosal interactions between bilateral S1 ^23,25^. Given that tSMS applied over the S1 has been reported to reduce the amplitudes of the N20 component^26^ and eHFOs^27^, it is plausible that tSMS may also influence transcallosal pathways mediating S1–S1 inhibition.

The purpose of the present study was to investigate the effects of tSMS applied over the S1 on the IHI between the bilateral S1, as assessed using SEPs and HFOs. We hypothesized that tSMS over the S1 would reduce IHI between the bilateral S1, as reflected by attenuated suppression of TS-evoked SEP components and HFOs induced by CS.

## Materials and methods

### Participants

A priori power analysis using G*Power (effect size = 0.25, α = 0.05, power = 0.80) indicated that a minimum sample size of nineteen participants was required. To account for possible dropouts, we recruited twenty-five right-handed healthy young adults (mean age ± SD = 22.2 ± 1.1 years; 12 males and 13 females). Handedness was assessed using the Edinburgh Handedness Inventory (mean ± SD, 92.8 ± 11.3; range, 57.8–100)^28^. Participants were screened to confirm the absence of neurological, psychiatric, cognitive, orthopedic, or cardiopulmonary disorders that could affect study outcomes. All participants provided written informed consent after receiving a full explanation of the study procedures. The study protocol was approved by the ethics committee of Aomori University of Health and Welfare (approval number: 240646) and was conducted in accordance with the principles of the Declaration of Helsinki. This trial was registered with the University Hospital Medical Information Network Clinical Trials Registry (UMIN-CTR) on 10/05/2024 (trial ID: UMIN000054358).

## Experimental procedure

**Fig. 1:**
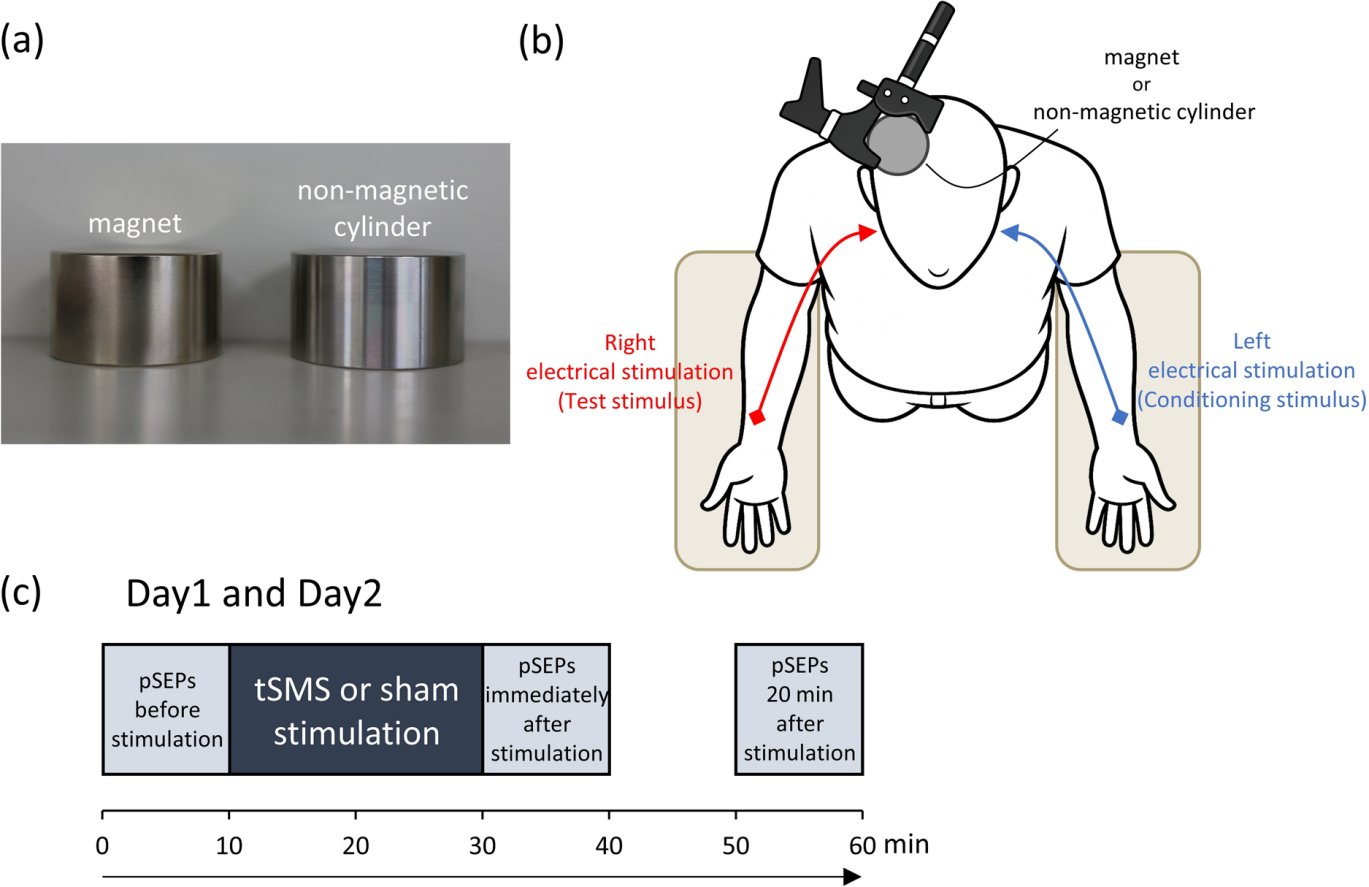
Experimental setup and procedure. (**a**) A cylindrical neodymium magnet (NdFeB; 50 mm diameter, 30 mm height; surface magnetic flux density, 534 mT; maximum energy product, 49 MGOe; attractive force, 862 N) was used for transcranial static magnetic stimulation (tSMS), whereas a visually matched, identically sized and weighted non-magnetic stainless-steel cylinder served as the sham device. (**b**) The magnet or sham cylinder was positioned over the primary somatosensory cortex (C4 according to the international 10–20 system) using an adjustable-arm light stand. Paired somatosensory evoked potentials (pSEPs) were elicited by electrical stimulation of the right and left median nerves. (**c**) pSEPs were recorded before, immediately after stimulation, and 20 min after stimulation.

The experimental setup was similar to that used in our previous study^27^. Participants were seated in a comfortable reclining chair equipped with a headrest. In the tSMS condition, a cylindrical neodymium magnet (NdFeB; diameter: 50 mm; height: 30 mm; surface magnetic flux density: 534 mT; maximum energy density: 49 MGOe; strength: 862 N) was used (NeoMag Co., Ltd., Chiba, Japan). In the sham condition, a non-magnetic stainless-steel cylinder matched in shape, weight, and appearance was applied (Fig. 1a). The device (tSMS or sham) was positioned over the right S1, corresponding to the C4 of the international 10–20 system, and held in place for 20 min using a movable arm-type light stand (C-stand, Avenger, Cassola, Italy) (Fig. 1b). pSEPs were recorded at three time points: before, immediately after, and 20 min after stimulation (Fig. 1c). This study employed a single-blind, randomized crossover study, with the order of stimulation conditions randomized across participants using a random number generator. Given that the effects of tSMS have been reported to be transient, typically lasting from several minutes to approximately 30 min after the end of stimulation, despite stimulation durations of 15–30 min^29,30^, the two sessions were scheduled on separate days with an interval of at least one week to minimize potential carryover effects.

## pSEP recording

**Fig. 2:**
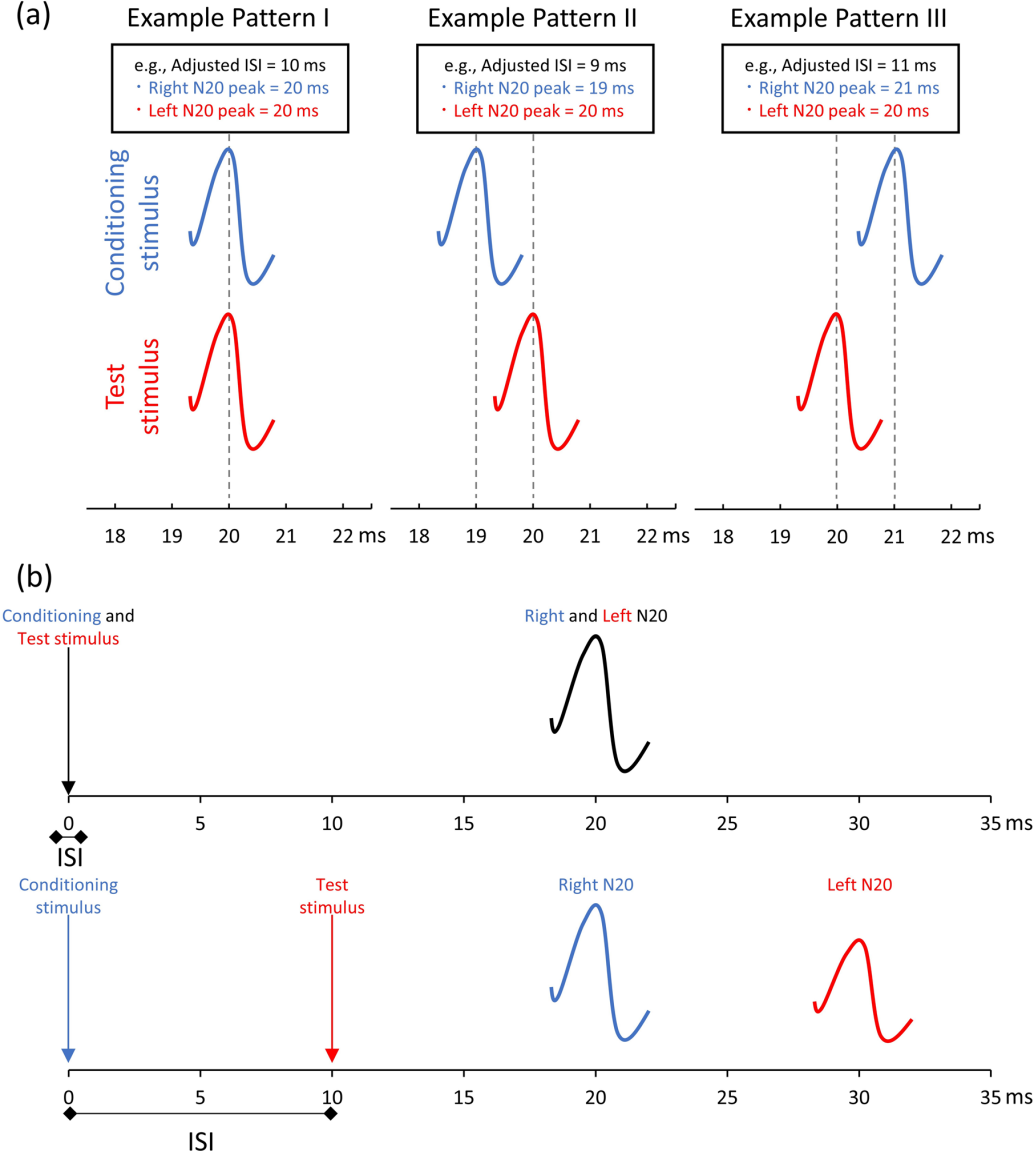
Interstimulus interval adjustment and recording conditions. (**a**) Interstimulus interval (ISI) between conditioning stimulus (CS) and test stimulus (TS) was adjusted to compensate for interhemispheric differences in N20 peak latencies. Three example patterns are shown to illustrate this adjustment procedure. Example Pattern I: When the right and left N20 peak latencies were equal (e.g., both 20 ms), the ISI was set to 10 ms. Example Pattern II: When the N20 latency evoked by CS (right N20 peak) preceded that evoked by TS (left N20 peak), the latency difference was subtracted from 10 ms (e.g., right: 19 ms; left: 20 ms → ISI = 9 ms). Example Pattern III: When the N20 latency evoked by TS (left N20 peak) preceded that evoked by CS (right N20 peak), the latency difference was added to 10 ms (e.g., right: 21 ms; left: 20 ms → ISI = 11 ms). (**b**) pSEPs were recorded under two conditions in a randomized order: a control condition with simultaneous bilateral median nerve stimulation (top) and an adjusted-ISI condition with a 10-ms ISI (bottom).

pSEPs were recorded using a paradigm in which stimulation of the left median nerve served as the CS and stimulation of the right median nerve as TS, following the protocol described by Norata et al. ^24^. The ISI was set to 10 ms, as this interval was reported to elicit the greatest IHI in the previous study, and adjusted for each participant based on individual N20 peak latencies^24^. Specifically, SEPs elicited by single right and left median nerve stimulations were first recorded to determine the N20 peak latencies for each side. The difference between these latencies was then subtracted from or added to the target ISI (10 ms) to obtain an individually adjusted ISI (detailed in Fig. 2a). After determining the adjusted ISI, pSEPs were recorded under two conditions in a randomized order: a control condition with simultaneous bilateral median nerve stimulation (ISI = 0 ms) and an adjusted-ISI condition with a 10-ms ISI between stimulations (Fig. 2b). Signals were recorded at a sampling rate of 10,000 Hz with a band-pass filter of 1–2000 Hz using a signal processor (Neuropack MEB-2300 system; Nihon-Kohden, Tokyo, Japan). Electrodes were placed at F1, F2, F3, F4, FC3, FC4, C3, C4, CP3, and CP4 according to the international 10–20 system. The ground electrode was placed on the forehead, and the reference electrode was attached to the left earlobe for right-hemisphere recordings and to the right earlobe for left-hemisphere recordings. Electrode impedance was maintained below 5 kΩ. Brief electrical pulses (0.2 ms) were delivered to the median nerves at a frequency of 4.7 Hz, with stimulus intensity gradually increased until a minimal visible twitch of the thenar muscles was observed (defined as the motor threshold), and subsequently set at 120% of this threshold. The same criterion was applied to both the left and right median nerve stimulations. At each time point (before, immediately after, and 20 min after stimulation), 1000 responses were recorded for each condition (control and adjusted-ISI conditions). Trials with amplitudes exceeding 100 µV were automatically rejected to eliminate artifact-contaminated data.

### Data analysis

**Fig. 3:**
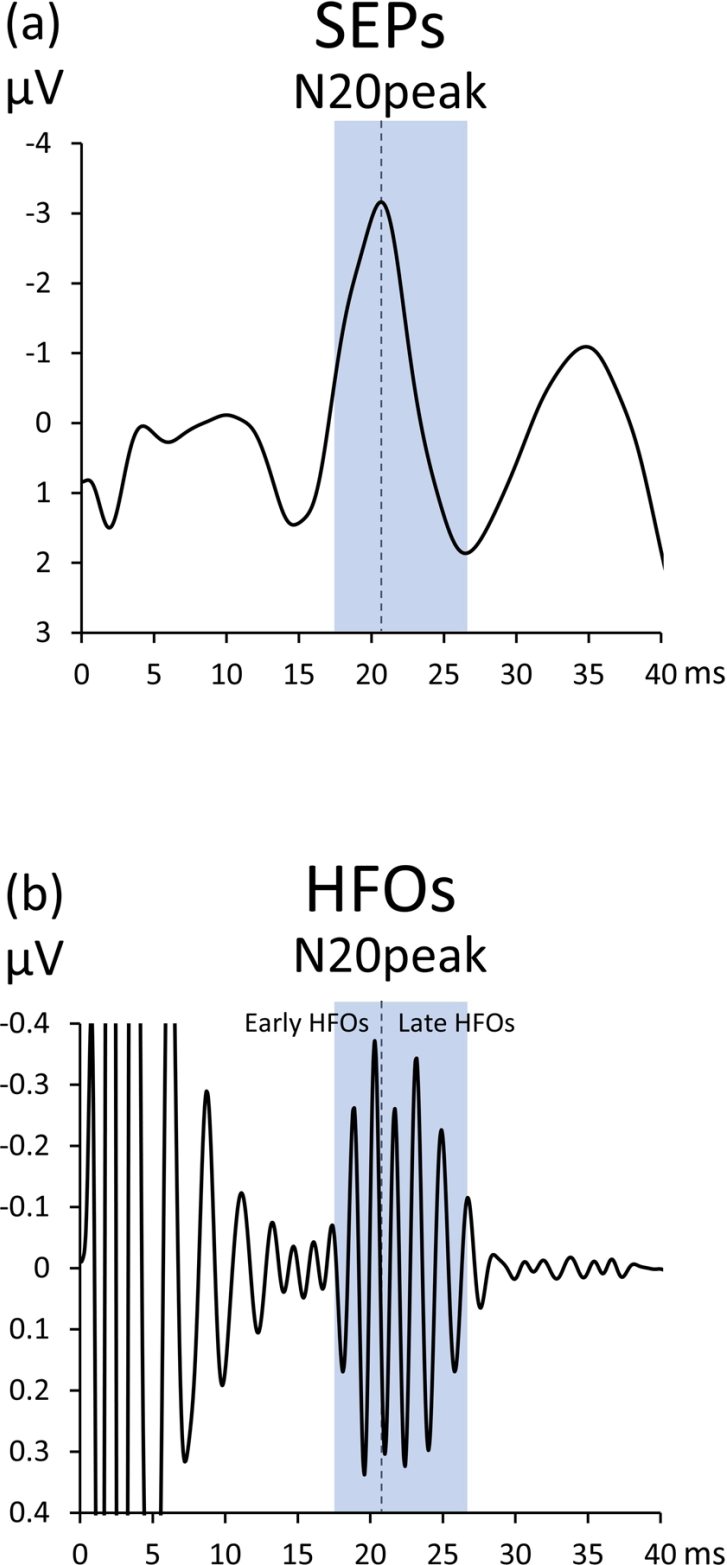
Representative waveforms. (**a**) Low-frequency somatosensory evoked potentials (SEPs) and (**b**) high-frequency oscillations (HFOs), subdivided into early (eHFOs) and late (lHFOs) components relative to the N20 peak. Blue-shaded areas indicate the eHFOs and lHFOs.

For each participant, 1000 responses were averaged per condition. SEP components were analyzed at CP3 after low-pass filtering at 300 Hz (Fig. 3a). The N20 onset-to-peak amplitude and the N20-P27 peak-to-peak amplitude (P27) were measured. HFOs were extracted from the CP3–F1 trace using band-pass filtering at 400–800 Hz, after confirming an out-of-phase relation between frontal and contralateral parietal signals^31^. HFOs were subdivided into eHFOs (N20 onset to N20 peak) and lHFOs (N20 peak to P27 peak) (Fig. 3b). After rectification, the area of each HFO was quantified.

### Statistical analysis

SEP amplitudes and HFO areas obtained in the adjusted-ISI condition were divided by the corresponding values in the control condition (i.e., ISI = 0 ms), and the resulting ratios were normalized to baseline values obtained before stimulation. Statistical analyses were performed using a two-way repeated-measures analysis of variance (ANOVA) with stimulation (tSMS vs. Sham) and time (before, immediately after, and 20 min after stimulation) as within-subject factors. Data normality was assessed using the Shapiro–Wilk test, and all variables included in the two-way repeated-measures ANOVA (N20, P27, total HFOs, eHFOs, and lHFOs) were log-transformed before analysis. Sphericity was assessed using Mauchly’s test, and when violated, the Greenhouse–Geisser correction was applied. Post hoc comparisons were conducted using Bonferroni correction to account for multiple comparisons. The level of statistical significance was set at α = 0.05.

## Results

All twenty-five participants completed the experiment without reporting any adverse effects. Table 1 summarizes the latencies and amplitudes of the N20 and P27 components, as well as the areas of HFOs, elicited by TS in the control (i.e., ISI = 0 ms) and adjusted-ISI (i.e., ISI = 10 ms) conditions. Table 2 presents the ratios calculated by dividing the amplitudes or areas in the adjusted-ISI condition by those in the control condition, with lower values indicating stronger inhibition. At baseline, the adjusted-ISI condition produced an approximately 20–30% reduction in N20 amplitude and a 10–20% reduction in HFO area relative to the control condition, indicating the presence of IHI. In contrast, the P27 amplitude increased by approximately 10% in the adjusted-ISI condition.

**Table 1:** SEP latencies/amplitudes and HFO areas.

Component	Stim	ISI		Pre	Post	Post 20 min
N20	tSMS	0 ms	Latency (ms)	20.98 ± 0.16	21.11 ± 0.17	21.12 ± 0.17
			Amplitude (µV)	2.74 ± 0.21	2.90 ± 0.20	2.91 ± 0.18
		10 ms	Latency (ms)	20.96 ± 0.17	21.08 ± 0.18	21.16 ± 0.16
			Amplitude (µV)	2.08 ± 0.19	2.14 ± 0.17	2.10 ± 0.15
	Sham	0 ms	Latency (ms)	21.02 ± 0.15	21.13 ± 0.14	21.32 ± 0.16
			Amplitude (µV)	2.89 ± 0.20	2.88 ± 0.18	2.80 ± 0.21
		10 ms	Latency (ms)	20.96 ± 0.15	21.21 ± 0.14	21.23 ± 0.16
			Amplitude (µV)	1.97 ± 0.19	2.05 ± 0.20	2.17 ± 0.19
P27	tSMS	0 ms	Latency (ms)	25.46 ± 0.32	25.54 ± 0.29	25.42 ± 0.30
			Amplitude (µV)	3.25 ± 0.33	3.31 ± 0.33	3.14 ± 0.31
		10 ms	Latency (ms)	26.34 ± 0.78	26.97 ± 0.79	26.62 ± 0.70
			Amplitude (µV)	3.38 ± 0.29	3.42 ± 0.31	3.36 ± 0.28
	Sham	0 ms	Latency (ms)	26.27 ± 0.72	27.13 ± 0.99	27.08 ± 0.95
			Amplitude (µV)	3.35 ± 0.32	3.39 ± 0.32	3.56 ± 0.36
		10 ms	Latency (ms)	26.31 ± 0.39	26.86 ± 0.76	26.95 ± 0.65
			Amplitude (µV)	3.40 ± 0.31	3.38 ± 0.31	3.41 ± 0.28
Total HFOs	tSMS	0 ms	Amplitude (µVཥms)	8.02 ± 0.78	8.60 ± 0.88	8.71 ± 0.83
		10 ms	Amplitude (µVཥms)	6.73 ± 0.61	7.94 ± 0.74	7.65 ± 0.72
	Sham	0 ms	Amplitude (µVཥms)	8.37 ± 0.78	8.81 ± 0.61	9.04 ± 0.69
		10 ms	Amplitude (µVཥms)	6.78 ± 0.57	7.34 ± 0.68	7.67 ± 0.66
Early HFOs	tSMS	0 ms	Amplitude (µVཥms)	3.53 ± 0.39	3.85 ± 0.42	3.87 ± 0.36
		10 ms	Amplitude (µVཥms)	2.80 ± 0.29	3.14 ± 0.32	3.25 ± 0.32
	Sham	0 ms	Amplitude (µVཥms)	3.79 ± 0.38	3.79 ± 0.33	3.98 ± 0.37
		10 ms	Amplitude (µVཥms)	2.81 ± 0.29	3.03 ± 0.30	3.24 ± 0.32
Late HFOs	tSMS	0 ms	Amplitude (µVཥms)	4.49 ± 0.46	4.74 ± 0.52	4.84 ± 0.53
		10 ms	Amplitude (µVཥms)	3.92 ± 0.39	4.80 ± 0.49	4.40 ± 0.48
	Sham	0 ms	Amplitude (µVཥms)	4.58 ± 0.46	5.02 ± 0.37	5.05 ± 0.39
		10 ms	Amplitude (µVཥms)	3.97 ± 0.35	4.31 ± 0.46	4.43 ± 0.41

ISI = interstimulus interval. Mean ± standard error.

**Table 2:** Ratios of SEP amplitudes and HFO areas.

Component	Stimulation	Pre	Post	Post 20 min
N20	tSMS	0.76 ± 0.05	0.81 ± 0.08	0.75 ± 0.05
	Sham	0.67 ± 0.05	0.70 ± 0.05	0.79 ± 0.05
P27	tSMS	1.10 ± 0.05	1.11 ± 0.07	1.16 ± 0.08
	Sham	1.07 ± 0.07	1.06 ± 0.07	1.05 ± 0.07
Total HFOs	tSMS	0.87 ± 0.04	0.96 ± 0.05	0.91 ± 0.06
	Sham	0.86 ± 0.04	0.83 ± 0.04	0.86 ± 0.05
Early HFOs	tSMS	0.87 ± 0.05	0.90 ± 0.08	0.92 ± 0.08
	Sham	0.74 ± 0.04	0.83 ± 0.06	0.83 ± 0.06
Late HFOs	tSMS	0.92 ± 0.06	1.08 ± 0.09	0.97 ± 0.09
	Sham	0.96 ± 0.06	0.84 ± 0.04	0.88 ± 0.06

Mean ± standard error.

## N20 amplitude

**Fig. 4:**
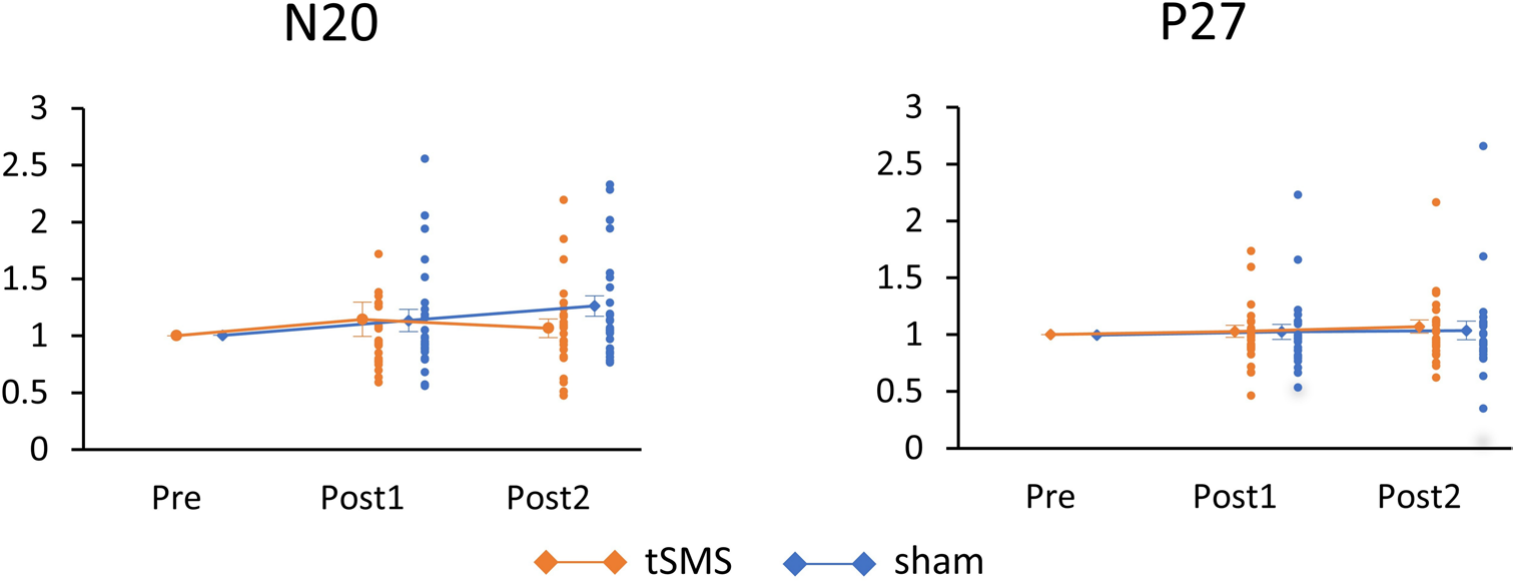
Amplitude ratios of N20 and P27. Mean amplitude ratios of N20 and P27 components of somatosensory evoked potentials (SEPs) recorded before (Pre), immediately after (Post1), and 20 min after (Post2) tSMS or sham stimulation. Data are normalized to values recorded before stimulation. Error bars represent the standard error of the mean. Orange and blue dots indicate individual data points for the tSMS and sham conditions, respectively.

Figure 4 (left plot) shows the results for the N20 amplitude ratio. A two-way repeated-measures ANOVA with factors of stimulation and time revealed no significant main effects of stimulation (F(1, 24) = 1.373, *p* = 0.253) or time (F(2, 48) = 0.954, *p* = 0.392), and no significant interaction between stimulation and time (F(2, 48) = 1.955, *p* = 0.153), suggesting that N20 amplitude ratios were comparable across stimulation conditions and time points.

## P27 amplitude

Figure 4 (right plot) shows the results for the P27 amplitude ratio. A two-way repeated-measures ANOVA with factors of stimulation and time revealed no significant main effects of stimulation (F(1, 24) = 0.428, *p* = 0.519) or time (F(2, 48) = 0.066, *p* = 0.937), and no significant interaction between stimulation and time (F(2, 48) = 0.407, *p* = 0.668), suggesting that N20 amplitude ratios were comparable across stimulation conditions and time points.

### Total HFOs

**Fig. 5:**
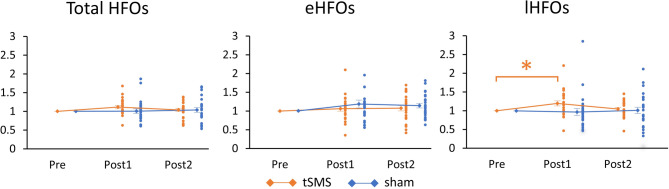
Area ratios of high-frequency oscillations. Mean area ratios of total, early and late high-frequency oscillations (Total HFOs, eHFOs and lHFOs) recorded before (Pre), immediately after (Post1), and 20 min after (Post2) tSMS or sham stimulation. Data are normalized to values recorded before stimulation. Error bars represent the standard error of the mean. Orange and blue dots indicate individual data points for the tSMS and sham conditions, respectively.

Figure 5 (left plot) shows the results for the total HFO area ratio. A two-way repeated-measures ANOVA with factors of stimulation and time revealed no significant main effects of stimulation (F(1, 24) = 2.297, *p* = 0.143) or time (F(2, 48) = 0.231, *p* = 0.795), and no significant interaction between stimulation and time (F(2, 48) = 1.980, *p* = 0.149), suggesting that N20 amplitude ratios were comparable across stimulation conditions and time points.

### eHFOs

Figure 5 (center plot) shows the results for the eHFO area ratio. A two-way repeated-measures ANOVA with factors of stimulation and time revealed no significant main effects of stimulation (F(1, 24) = 1.620, *p* = 0.215) or time (F(2, 48) = 0.730, *p* = 0.487), and no significant interaction between stimulation and time (F(1.491, 35.783) = 0.346, *p* = 0.647), suggesting that N20 amplitude ratios were comparable across stimulation conditions and time points.

### lHFOs

Figure 5 (right plot) shows the results for the lHFO area ratio. A two-way repeated-measures ANOVA with factors of stimulation and time revealed a significant main effect of stimulation (F(1, 24) = 4.618, *p* = 0.042), no significant main effect of time (F(2, 48) = 0.297, *p* = 0.744), and a significant interaction between stimulation and time (F(2, 48) = 4.595, *p* = 0.015). To further examine this interaction, one-way repeated-measures ANOVAs were conducted separately for each stimulation condition (tSMS and sham) across three time points (baseline, immediately after, and 20 min after stimulation). In the tSMS condition, there was a significant main effect of time (F(1.584, 38.023) = 5.591, *p* = 0.012), whereas no significant main effect was observed in the sham condition (F(2, 48) = 1.120, *p* = 0.335). Post hoc pairwise comparisons in the tSMS condition indicated that the lHFO area ratio significantly increased from baseline to immediately after stimulation (*p* = 0.030), with no significant differences between baseline and 20 min after stimulation or between immediately after and 20 min after stimulation (*p* > 0.05).

## Discussion

This study investigated the effects of tSMS applied over the S1 on IHI from the stimulated to the non-stimulated S1. To this end, we examined how tSMS modulated CS-induced inhibition of TS-evoked SEPs and HFOs using a pSEPs paradigm. The results indicated differential effects of tSMS across these components. Specifically, tSMS significantly increased the lHFO area ratio, indicating an attenuation of CS-induced inhibition of lHFOs. In contrast, no significant changes were observed in either the N20/P27 component or eHFOs. These findings provide new insights into how tSMS may influence interhemispheric interactions within the somatosensory system.

The pSEPs methodology was adopted from a previous study demonstrating IHI for the N20 and P25 (P27 in the present study) components as well as HFOs^24^. However, whereas IHI was evident for N20 and HFOs, it was not observed for P27 (Table 2). SEP components occurring after N20, but not N20 itself, are known to be influenced by widespread projections from non-specific thalamic nuclei to superficial cortical layers^32^, which are closely related to arousal regulation^33^. Indeed, the amplitudes of P27 and subsequent SEP components decrease with shorter ISIs^34^, likely reflecting habituation, while directing attention to the target stimulus enhances their amplitudes^35^. It is therefore possible that CS-induced activation of non-specific thalamic nuclei, potentially related to transient arousal, exerted bilateral modulatory influences that mitigated transcallosal inhibition, thereby attenuating or obscuring IHI specifically for the P27 component. Nonetheless, this interpretation remains speculative and warrants further investigation to elucidate the underlying mechanisms.

Regarding the effects of tSMS, previous studies have shown that tSMS not only reduces cortical excitability in the cortex directly beneath the magnet but also modulates activity within distributed cortical networks^18,27,36–47^. Several cellular-level mechanisms have been proposed to account for these effects, although they remain inconclusive. The main hypotheses include: (a) static magnetic field (SMF)-induced reorientation of membrane phospholipids due to their diamagnetic anisotropy^48^, (b) changes in membrane surface tension arising from magnetic pressure^49^, and (c) Lorentz forces acting on ions within neuronal membrane channels^50^. On the other hand, some animal studies have suggested that the effects of tSMS may be transient and state-dependent, rather than reflecting stable cellular mechanisms^51^.

In the present study, tSMS did not significantly affect the amplitude ratios of the N20 and P27 components. This finding contrasts with previous reports showing that 1-Hz rTMS over the S1 increased the N20–P27 amplitude in the contralateral S1 in healthy adults^20^. One possible explanation is the high stability of the N20 component, which reflects neural activity in Brodmann area 3b of the S1. The N20 is known to be highly reproducible across experimental conditions and relatively insensitive to changes in physiological states, such as wakefulness, light sleep, or light anesthesia^52,53^. Another factor may relate to the choice of ISI. Norata et al. ^24^ reported that IHI between bilateral S1 occurs at ISIs ranging from 5 to 40 ms, with maximal inhibition of both N20 and P25 observed at an ISI of 10 ms. In contrast, Ragert et al. ^54^ and Brodie et al. ^23^ demonstrated significant inhibitory effects at longer ISIs of 15–35 ms, whereas Ishii et al. ^22^ found no significant IHI across a wide range of ISIs (1–100 ms). At present, there is no consensus regarding the optimal ISI for reliably eliciting IHI within the S1. Given these heterogeneous findings, the lack of tSMS-induced modulation of the N20 component observed in the present study may be attributable, at least in part, to the use of a 10-ms ISI, highlighting the need for future studies to systematically examine the effects of tSMS across different ISIs.

Consistent with the findings for the N20 and P27 components, tSMS did not significantly affect the area ratios of eHFOs, which are thought to reflect action potentials of thalamocortical fibers projecting to the S1 ^55^. In a previous study by Norata et al. ^24^, the CS significantly suppressed TS-evoked total HFOs and lHFOs, whereas suppression of eHFOs was relatively weak and not significant across a range of ISIs (5, 10, 20, and 40 ms). This pattern suggests that interhemispheric interactions involving thalamocortical afferents are considerably weaker than those mediated by other circuits. Consequently, the absence of a tSMS-induced modulation of eHFO-related IHI in the present study likely reflects the limited engagement of thalamocortical pathways in this form of transcallosal interaction.

TSMS applied over the S1 significantly attenuated CS-induced inhibition of lHFOs, which are considered to reflect the activity of GABAergic interneurons within area 3b of the S1 ^55^, a form of intracortical processing. It should be noted that, although lHFOs have been associated with GABAergic interneuron activity, they cannot be regarded as a specific marker of such activity, and their underlying mechanisms remain indirect^56,55,57^. Callosal projection neurons are primarily pyramidal cells located in layers II/III and V that use glutamate or aspartate as neurotransmitters^58^. Callosal inputs form direct excitatory connections onto cortical pyramidal cells and can also evoke polysynaptic inhibition via the recruitment of local GABAergic interneurons^59,60^. IHI is thought to arise, at least in part, from such polysynaptic inhibitory mechanisms^60,61^. Accordingly, activation of the transcallosal pathway from the right S1 by left median nerve stimulation (CS) may recruit GABAergic interneurons in the contralateral S1, thereby influencing intracortical inhibitory processing and thus lHFOs evoked by right median nerve stimulation (TS). Within this framework, the observed attenuation of CS-induced suppression of lHFOs by tSMS may reflect a reduction in transcallosal influences on intracortical inhibitory circuits, resulting in altered excitation–inhibition balance within the local S1 microcircuit. However, this interpretation should be treated with caution. Although the interaction between conditioning and test stimuli argues against a simple global change in cortical excitability, such a contribution cannot be fully excluded. Related evidence from studies of IHI within the M1 further supports the notion that the interhemispheric effects of NIBS depend on both the stimulation protocol and the cortical network under investigation. For example, continuous theta burst stimulation applied over one M1, which reduces cortical excitability, has been shown to increase excitability in the contralateral M1 while concurrently reducing short-interval intracortical inhibition (SICI), a process largely mediated by GABAergic mechanisms^62^. In contrast, 1-Hz rTMS over the M1, which also reduces cortical excitability, has been reported to increase contralateral motor-evoked potential amplitudes without affecting SICI^63^. Taken together, these findings, along with the present results, indicate that the impact of NIBS on interhemispheric interactions is strongly influenced by stimulation parameters and the intrinsic physiological characteristics of the targeted cortical area. Further studies are required to deepen our understanding of how NIBS, including tSMS, modulates transcallosal inhibition.

This study has several limitations. First, because all participants were healthy young adults, the findings may not generalize to clinical populations. Second, only a single stimulation duration and magnetic field strength were tested; thus, future studies should systematically vary these parameters to identify optimal conditions. Third, because we examined only a single ISI (10 ms), it remains unclear how ISI influences the modulation of SEPs and HFOs. Finally, the paired SEP paradigm does not provide a direct measure of interhemispheric inhibition; thus, the present findings should be interpreted with caution.

In conclusion, the present study suggests that tSMS modulates interhemispheric interactions within the S1. Specifically, tSMS applied over the S1 attenuated interhemispheric suppression of lHFOs in the contralateral S1, whereas eHFOs and early SEP components were unchanged. These findings are consistent with the possibility that tSMS may influence transcallosal interactions involving intracortical inhibitory processing, rather than thalamocortical afferent pathways. Overall, tSMS may represent a useful tool for probing and potentially modulating sensory interhemispheric interactions.

## Data Availability

The datasets used during the study will be available from the corresponding author on reasonable request.
